# The complete mitochondrial genome of *Gruberia lanceolata* (Gruber, 1884) Kahl, 1932 (Ciliophora: Heterotrichea)

**DOI:** 10.1080/23802359.2019.1674199

**Published:** 2019-10-09

**Authors:** Mi-Hyun Park, Gi-Sik Min

**Affiliations:** Department of Biological Sciences, Inha University, Incheon, The Republic of Korea

**Keywords:** *Gruberia lanceolata*, heterotrichean ciliate, new class of mitogenome, South Korea

## Abstract

The ciliate *Gruberia lanceolata* (Gruber, 1884) Kahl, 1932 belonging to the class Heterotrichea was sampled from the coastal waters of South Korea. The complete mitogenome in its linear form and large size (∼40 kb) was obtained. It consisted of 27 protein-coding genes (PCGs), two ribosomal subunit RNA (rRNA) genes, four transfer RNAs (tRNAs), and ten unclassified open reading frames (ORFs). Their telomeric structures were capped, with repeat regions at both ends. We analyzed its phylogenetic tree using the data of its respiratory chain complex I genes. It can be suggested that the complete mitochondrial genome of *G. lanceolata* can be recorded as a new class of the mitogenome.

To date, mitogenomes of only 12 species have been identified in members belonging to the phylum Ciliophora (Pritchard et al. 1990; Burger et al. 2000; de Graaf et al. 2009, 2011; Swart et al. 2012; Li et al. 2018; Park et al. 2019). The genus *Gruberia* is not well-known and comprises only of seven species (Chen et al. 2018) in the class Heterotrichea. It was sampled from the coastal waters of the Seonnyeobawi Beach, Yellow Sea, Korea (37°26′N, 126°22′E; salinity: 29 psu), on 24 October 2014. A slide of protargol-impregnated specimens was deposited at the National Institute of Biological Resources, Korea (NIBRPR0000106616) (Park and Min 2016). The mitochondrial DNA was sequenced using the Illumina Miseq platform (Macrogen, Seoul, Korea) and the mitogenome from partial *cox1* was assembled using NOVOplasty 2.6.3 (Dierckxsens et al. 2016). Furthermore, we determined complete mitogenome sequence by comparing it with the contigs available in SOAPdenovo v2.01 (Luo et al. 2012). The annotation was performed using MFANNOT webserver (http://megasun.bch.umontreal.ca/cgi-bin/mfannot/mfannotInterface.pl) and RNAweasal webserver (http://megasun.bch.umontreal.ca/cgi-bin/RNAweasel/RNAweaselInterface.pl) based on ERPIN version 5.2.1. (Gautheret and Lambert 2001), with assistance from the mold mitochondrial genetic code (i.e. genetic code 4). The transfer RNA-encoding genes were identified using the tRNAscan-SE (Lowe and Eddy 1997; Lowe and Chan 2016). The maximum-likelihood (ML) phylogenetic tree for concatenated mitochondrial complex I genes (*nad1*, *nad2*, *nad3*, *nad4*, *nad5*, *nad7*, *nad9*, and *nad10*; except *nad4L* and *nad 6*) was constructed using RAxML (Stamatakis 2014) with 1000 bootstrap replicates based on the amino acids aligned using MAFFT (Katoh and Standley 2013). All gaps representing many contiguous non-conserved positions were removed using the Gblocks Server Version 0.91 b (Talavera and Castresana 2007).

The complete mitochondrial genome of *Gruberia lanceolata* consisting of 39,989 bp was deposited in the GenBank (accession number MK301177) for identification. The mitogenome is a linear structure comprising repeat regions in the telomeres at both ends, and its transcription occurs in single, as well as reverse directions. The nucleotide composition of *G. lanceolata* is as follows: A: 44.2%, C: 12.3%, G: 8.1%, T: 35.4%. The GC content of the complete genome was found to be 20.4%. The genome contained 27 protein-coding genes (PCGs), 2 ribosomal subunit RNA (rRNA) genes, 4 tRNAs, and 10 unclassified open reading frames (ORFs). The 27 PCGs included genes encoding the following molecules: respiratory chain complex I (*nad1*, *nad2*, *nad3*, *nad4*, *nad4*_*i*, *nad4L*, *nad5*, *nad6*, *nad7*, *nad9*, and *nad10*), complex III (*cob*), complex IV (*cox1* and *cox2*), and complex V (*atp9*); cytochrome c-related genes (*ccmf*_*i* and *ccmf*_*ii*); small and large ribosomal subunit genes (*rps3*, *rps4*, *rps8*, *rps12*, *rps14*, *rps19*; *rpl2*, *rpl6*, *rpl14*, and *rpl16*).

Based on the data of the complex I genes, it was determined that the species were clustered within their own classes, and *G. lanceolata* formed a separate clade at the class level in all gene trees (Figure 1). It can be expected that the phylogenetic analysis using the complete mitogenome well represents the phylogenetic relationship of ciliates.

**Figure 1.**
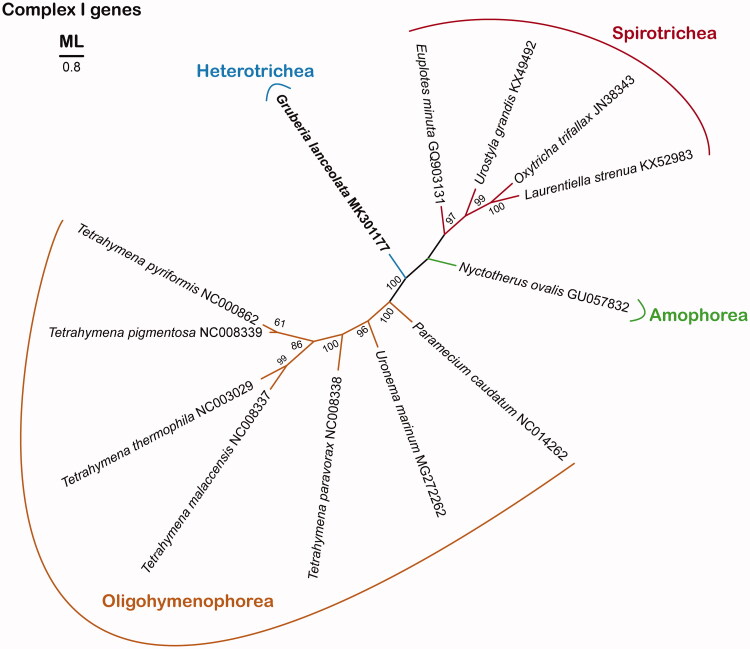
The maximum-likelihood (ML) tree for mitochondrial respiratory chain complex I (*nad1*, *nad2*, *nad3*, *nad4*, *nad5*, *nad7*, *nad9*, and *nad10*) genes. The ML tree of complex I genes is unrooted.
